# Impact of Statins on Gene Expression in Human Lung Tissues

**DOI:** 10.1371/journal.pone.0142037

**Published:** 2015-11-04

**Authors:** Jérôme Lane, Stephan F. van Eeden, Ma’en Obeidat, Don D. Sin, Scott J. Tebbutt, Wim Timens, Dirkje S. Postma, Michel Laviolette, Peter D. Paré, Yohan Bossé

**Affiliations:** 1 Institut universitaire de cardiologie et de pneumologie de Québec, Quebec City, Canada; 2 University of British Columbia, Department of Medicine & Center for Heart Lung Innovation, St. Paul’s Hospital, Vancouver, Canada; 3 The PROOF Centre of Excellence, Vancouver, Canada; 4 University of Groningen, University Medical Center Groningen, GRIAC research institute, Groningen, The Netherlands; 5 Department of Molecular Medicine, Laval University, Quebec City, Canada; University Hospital Freiburg, GERMANY

## Abstract

Statins are 3-hydroxy-3-methylglutaryl-coenzyme A reductase inhibitors that alter the synthesis of cholesterol. Some studies have shown a significant association of statins with improved respiratory health outcomes of patients with asthma, chronic obstructive pulmonary disease and lung cancer. Here we hypothesize that statins impact gene expression in human lungs and may reveal the pleiotropic effects of statins that are taking place directly in lung tissues. Human lung tissues were obtained from patients who underwent lung resection or transplantation. Gene expression was measured on a custom Affymetrix array in a discovery cohort (n = 408) and two replication sets (n = 341 and 282). Gene expression was evaluated by linear regression between statin users and non-users, adjusting for age, gender, smoking status, and other covariables. The results of each cohort were combined in a meta-analysis and biological pathways were studied using Gene Set Enrichment Analysis. The discovery set included 141 statin users. The lung mRNA expression levels of eighteen and three genes were up-regulated and down-regulated in statin users (FDR < 0.05), respectively. Twelve of the up-regulated genes were replicated in the first replication set, but none in the second (p-value < 0.05). Combining the discovery and replication sets into a meta-analysis improved the significance of the 12 up-regulated genes, which includes genes encoding enzymes and membrane proteins involved in cholesterol biosynthesis. Canonical biological pathways altered by statins in the lung include cholesterol, steroid, and terpenoid backbone biosynthesis. No genes encoding inflammatory, proteases, pro-fibrotic or growth factors were altered by statins, suggesting that the direct effect of statin in the lung do not go beyond its antilipidemic action. Although more studies are needed with specific lung cell types and different classes and doses of statins, the improved health outcomes and survival observed in statin users with chronic lung diseases do not seem to be mediated through direct regulation of gene expression in the lung.

## Introduction

Statins block HMG-CoA reductase (HMGCR), a rate-limiting enzyme responsible for the synthesis of endogenous cholesterol and non-sterol isoprenoids. Statins are used predominantly to manage hypercholesterolemia and for secondary prevention to reduce the risk of cardiac events [1, 2]. Furthermore, statins inhibit cholesterol-independent pathways leading to secondary or pleiotropic actions such as antioxidant [3] and anti-inflammatory [4–6] effects. In humans, statin treatment has been associated with improved survival in patients with lung cancer [7–10], fewer acute COPD and asthma exacerbations [11–17], reduced risk of pulmonary hypertension [18, 19], reduced rate of lung function decline [20, 21] and decreased all-cause mortality in COPD patients, mostly in retrospective studies [22–27]. However, a recent study demonstrated no effect of statin on exacerbation rates and the time to a first exacerbation in patients with moderate-to-severe COPD [28]. In the later study, it was unclear whether the lack of clinical benefit would apply to patients with less severe COPD.

Many hypotheses were put forward to explain the clinical benefits of statins on chronic lung diseases [29]. Better outcomes in patients treated with statins may be simply mediated by the indirect effects on cardiovascular comorbidities. However, statin-induced reduction of systemic inflammation is believed as the most likely explanation underlying the benefits of statins in lung diseases. Supporting this hypothesis was the greatest increase in exercise tolerance in COPD patients with a better lowering effect of statins on systemic inflammation [30]. Statins were also more beneficial in COPD patients with high baseline level of systemic inflammation [31]. In cigarette smoking-induced emphysema rat models, simvastatins were shown to prevent airway inflammatory infiltration [32, 33]. Accordingly, not only systemic inflammation, but also pulmonary inflammation may be attenuated in statin users. Simvastatin was also shown to prevent anatomical COPD lesions (e.g. enlargement of airspaces, small airway thickening) in these rat models and to counteract the induction of MMP9 activity and mRNA expression levels of *TGFB1* and *CTGF* in lung tissue, suggesting a direct role of statins in the lung. Similarly, in a chronic guinea pig smoking model, simvastatin was shown to prevent smoke-induced pulmonary hypertension and vascular remodeling as well as to reverse smoke-induced endothelial dysfunction and emphysema [34]. In human lung fibroblasts, statins were shown to inhibit TGFβ1-induced fibronectin and CTGF expression as well as to inhibit cytokine-induced release of matrix metalloproteinases [35–37]. Animal and cell models are thus supporting the direct actions of statins in the lung. Whether these effects are observed in the lung of patients treated with standard cardio-protective dose of statins is unknown. It is also unclear whether the effects of statins are mediated, or at least measurable, through direct regulation of gene expression in human lung. This calls for a genomic approach given the pleiotropic nature of statins and expected regulation of genes encoding proteases, fibrogenic, inflammatory, and growth factors.

In human primary hepatocytes, atorvastatin and rosuvastatin were shown to modulate 128 and 869 genes in common after 24 and 48 hours of treatment, respectively [38]. This genome-wide gene expression study confirmed the role of statins in modulating genes involved in hepatic cholesterol homeostasis, but also identified other genes implicated in a variety of pathways that may explain the pleiotropic and secondary adverse effects of statins. We hypothesized that some of the clinical benefits of statins on chronic lung diseases are related to their pleiotropic properties and can be detected by measuring gene expression in human lung tissues. The aim of this study was to evaluate the impact of statin treatment on gene expression in human lung in order to identify novel molecular pathways underpinning the potential benefits of statins in chronic lung diseases.

## Materials and Methods

### Study participants

Lung parenchymal tissues were obtained from patients undergoing lung resection for peripheral lung nodules/cancer between 2004 and 2008 at the oncology clinic of the *Institut universitaire de cardiologie et de pneumologie de Québec* (IUCPQ) [39]. Tissues were obtained from macroscopically normal appearing lung regions away from the tumor or tumor margins. Henceforth, this dataset is referred to as the Laval or discovery cohort. Two replication cohorts were collected at the University of British Columbia (UBC) and the University of Groningen. At UBC, the majority of samples were from patients undergoing resection of small peripheral lung lesions. Additional samples were explanted lungs from lung transplantation. At Groningen, the lung specimens were obtained at surgery from patients with various lung diseases, including patients undergoing therapeutic resection for lung tumors and lung transplantation. For the discovery and the two replication sets, selection of patients was based on tissue availability. Patients with missing information on statin use were excluded. All patients were of white European descent confirmed by whole-genome genotyping on the Illumina Human1M-Duo BeadChip. The primary indication for taking statin was to lower blood cholesterol levels and reduce the risk of cardiac events. No patients were prescribed statin specifically for lung diseases. Preoperatively, patients underwent pulmonary function testing in which lung volumes, forced expiratory volume in 1 sec (FEV_1_) and forced vital capacity (FVC) were determined. COPD was defined based on spirometry as per the GOLD recommendations [40]. Primary diagnostic and lung cancer histology were obtained from the pathology report. Smoking history included self-reported smoking status and number of pack-years. Statin use was abstracted from the patients’ medical records.

### Ethics statements

At Laval, lung specimens were collected from patients undergoing lung cancer surgery and stored at the IUCPQ site of the Respiratory Health Network Tissue Bank of the “Fonds de recherche du Québec–Santé” (www.tissuebank.ca). Written informed consent was obtained from all subjects and the study was approved by the IUCPQ ethics committee. At Groningen, lung specimens were provided by the local tissue bank of the University Medical Center Groningen (Department of Pathology, www.umcg.nl/EN/corporate/pages/default.aspx) and the study protocol was consistent with the Research Code of the University Medical Center Groningen (www.umcg.nl/en/research/researchers/general/researchcode/pages/default.aspx) and Dutch national ethical and professional guidelines (“Code of conduct; Dutch federation of biomedical scientific societies”; http://www.federa.org). At Vancouver, the lung specimens were provided by the Centre for Heart Lung Innovation Biobank at St Paul's Hospital and subjects provided written informed consent. The study was approved by the ethics committees at the University of British Columbia-Providence Health Care Research Institute Ethics Board.

### Lung tissue processing

For the discovery set, lung specimens were surgically explanted and immediately examined by a pulmonary pathologist. After processing for pathologic diagnosis and staging, a nonneoplastic pulmonary parenchyma sample (2–5 cm^3^) was harvested from a site as far distant as possible from the tumor. The research specimens were immediately divided into smaller fragments (~0.5 cm^3^) placed in 5-mL cryovials and snap-frozen in liquid nitrogen. The cryovials were then transported in dry ice to the IUCPQ Tissue Bank where they were stored at -80°C until further processing.

For UBC replication set, immediately following resection, the lung or lobe was obtained from the operating or autopsy room. After the clinical specimens of the lesion, lymph nodes and the resection margin were obtained, the lobes and lungs were inflated using a 50% mixture of CryomatrixR and saline and frozen in liquid nitrogen fumes. The frozen lungs and lobes were then cut into 7–15 two cm thick slices using a band saw and multiple randomly stratified blocks were acquired (1-3/slice) using a power driven hole saw fitted with a 1.5 cm diameter bit. The frozen “cores” were stored at -80°C for later RNA extraction. For the Groningen replication set, immediately following resection, the lung or lobe was obtained from the operating room and processed for pathological diagnosis and staging. After this procedure a non-neoplastic pulmonary parenchyma sample (2–5 cm^3^) was harvested from a site distant from the tumor. The research specimens were then divided into smaller fragments (~1 cm^3^), snap-frozen in liquid isopentane, and stored at -80°C.

### Whole-genome gene expression

Total RNA from whole lung specimens was extracted using the SV96 Total RNA Isolation System (Promega). Lung mRNA samples from each patient were hybridized on a custom Affymetrix array (GEO platform GPL10379) and expression data are available through GEO23546. All statistical analysis was performed with R statistical software version 3.1.1 and Bioconductor packages [41]. Standard quality controls were applied to remove outliers as we described previously [39]. Gene expression was quantile-normalized [42] and summarized by Robust Multi-array Average (RMA) [43, 44] using the rma function as implemented in the *affy* package.

### Genes differentially expressed in the discovery set

Linear regressions on gene expression traits were performed in the discovery cohort (Laval). Expression traits were adjusted for age, gender and smoking status. The functions lmFit, eBayes and topTable implemented in the *limma* package were used to identify genes differentially expressed between patients who were or were not treated with statins. A total of 52,378 probe sets were tested for association with statin usage in the discovery cohort. The Benjamini-Hochberg (BH) procedure and Bonferroni correction were applied to correct for multiple testing.

Applying these analyses in the discovery cohort yielded a test statistic that deviated from expected distribution based on a quantile-quantile (QQ) plot, which provides a visual summary of the distribution of the observed p values generated by the genome-wide gene expression experiment. Therefore, surrogate variable analysis (SVA) [45] was performed to remove unwanted and unknown sources of variation in the data. Surrogate variables were detected using the sva function implemented in the *sva* package. Adjustment for covariates was performed by the lmFit function and results were computed using the eBayes and topTable functions as described above.

### Replication of genes differentially expressed

Genes significantly differentially expressed with a false discovery rate (FDR) lower than 0.05 in the discovery cohort were tested for validation in the two replication cohorts (UBC and Groningen). Linear regressions were performed as described above in Groningen and UBC sets individually. To increase sample size, replication analyses were also performed by combining the two cohorts. The two cohorts were combined using the ComBat adjustment method [46] in order to take into account the differences that exist in the clinical characteristics of patients as well as lung tissues collection and processing methods between the two sets. Finally, a meta-analysis combining the discovery and replication sets was also performed using the Fischer’s method combining p-values derived from the three individual cohorts [47]. The function fischer.method from the *MADAM* package was applied [48]. Genes were considered significantly replicated if they had a p-value < 0.05 in at least one of the replication set, in the combined Groningen-UBC set, or the meta-analysis. S1 Fig illustrates an overview of the analytical steps.

### Pathway analysis

Biological pathways were studied with the Gene Set Enrichment Analysis (GSEA) program [49]. Analysis was performed using the molecular signatures database MSigDB version 4.0. Annotated genes were pre-ranked based on t statistics testing differential expression between patients taking or not taking statins. Gene sets with a False Discovery Rate (FDR) q-value lower than 0.05 were considered statistically significant. Canonical pathways from REACTOM [50] and KEGG [51] were further studied.

### Sub-analysis without patients with severe-to-very-severe COPD

The role of statin treatment in COPD is controversial [11, 20, 21, 28]. Therefore, a sub-group analysis was performed by analyzing patients without severe COPD by excluding COPD patients with a post-bronchodilator FEV_1_ of < 50% of predicted (GOLD stages 3 and 4). Pre-bronchodilator values were taken if post-bronchodilator were not available. All steps used in the main analysis were also applied to this sub-analysis.

### Quantitative real-time PCR (qPCR)

qPCR was used to validate the expression of 12 genes differentially expressed between patients taking or not taking statins. Lung parenchymal tissues were obtained from the IUCPQ biobank. Twenty statin users were selected and matched with non-statin users for gender, age, smoking status (years since smoking cessation for former smokers) and lung cancer histology. These samples were not included in the Laval discovery set. The clinical characteristics of patients used in the qPCR experiment are shown in S1 Table. RNA was extracted from 30 mg of frozen lung tissue using the RNeasy Universal Plus Mini kit (Qiagen). RNA concentration and purity was assessed by UV 260/280 nm ratio with the NanoVue spectrophotometer (GE Healthcare). Two micrograms of RNA were converted to cDNA using Quantitect Reverse Transcription kit (Qiagen). qPCR was performed using the SsoAdvanced Universal SYBR Green Supermix (Bio Rad) on the Bio Rad CFX384 Real-time PCR system. Cycling steps were 1 cycle of 30 sec at 95°C then 40 cycles of 15 sec at 95°C and 30 sec at annealing/elongation temperature. Two genes (EBP and TM7SF2) were amplified using 5% formamide and a touchdown cycling program consisting of 1 cycle of 30 sec at 95°C then 18 cycles of 15 sec at 95°C and 15 sec at 69°C minus 0.5°C/cycle and 15 sec at 60°C then 30 cycles of 15 sec at 95°C and 30 sec at 60°C. Three reference genes were considered including *GAPDH*, *ACTB* and *B2M*. The primers were designed using the software Primer3 v.0.4.0 (http://frodo.wi.mit.edu/primer3) and synthesized by Integrated DNA Technologies (Toronto, Ontario). PCR primers were tested *in silico* using BLAT in UCSC (http://genome.ucsc.edu/index.html) to confirm their binding to a unique region of the human genome (hg38) and the absence of underlying polymorphism. Primers for target and reference genes, amplicon sizes, and annealing temperatures are shown in S2 Table. For each gene, the experimental samples were tested in triplicate. The cDNA copy numbers of each sample were calculated according to the standard curve method and normalized to the average copy number of the three reference genes. The fold changes were obtained by dividing mean copy numbers of cDNA between the two groups (i.e. statin users compared to non-statin users). One-sided paired t-tests were used to assess significant differences in gene expression between statin and non-statin users.

## Results

### Discovery set

Whole-genome gene expression data were obtained from 479 patients. A total of 408 patients had information on statin use, were of white European ancestry, and passed all quality controls. The demographic and clinical features are summarized in Table 1. Patients underwent lung cancer surgery predominantly for adenocarcinoma (n = 235) and squamous cell carcinoma (n = 103). One hundred and forty-one patients (34.6%) were using statins. Most statin users (76.6%) were prescribed lipophilic statins. A larger proportion of women than men were on statin therapy. COPD was present in 56.1% of patients. There was no significant difference in statin usage between patients with or without COPD. Age, body mass index and pack-years were significantly higher in patients taking statins. Lung function measured by FEV_1_% predicted post-bronchodilator was similar between patients taking or not taking statins (p = 0.808), but the FEV_1_/FVC ratio tended to be higher in the statin group (p = 0.045) (Table 1).

**Table 1:** Clinical characteristics of the discovery cohort according to statin treatment.

	Statins			
	Non-users (n = 267)	Users (n = 141)	Total (n = 408)	p-values
Statin types ^(1)^				
Atorvastatin		83	108 (lipophilic)	
Simvastatin		22		
Lovastatin		2		
Fluvastatin		1		
Pravastatin		14	32 (hydrophilic)	
Rosuvastatin		18		
Gender				
Men	134	46	180	9.92E-04^(2)^
Women	133	95	228	
Smoking status				
Never	27	9	36	NS^(2)^
Former	179	103	282	
Current	61	29	90	
Chronic Obstructive Pulmonary Disease				
NAs	25	9	34	NS^(2)^
no	105	59	164	
yes	137	73	210	
Age (mean ± SD)	61.74 ± 10.33	66.44 ± 8.24	63.37 ± 9.93	1.05E-06^(3)^
BMI (mean ± SD)	26.21 ± 5.21	27.62 ± 5.37	26.27 ± 5.30	0.01^(3)^
Pack-years (mean ± SD:NAs)	41.83 ± 29.27:23	48.30 ± 30.17:14	44.04 ± 30.17:37	0.049^(3)^
Primary diagnosis				
Lung cancer	266	141	407	
Adenocarcinoma	152	83	235	
Squamous cell carcinoma	62	41	103	
Others	52	17	69	
COPD	1	0	1	
Lung function				
FEV_1_% predicted, post-BD	82.4 ± 17.8 [7]	81.9 ± 16.8 [3]	82.2 ± 17.4 [10]	0.808^(3)^
FVC % predicted, post-BD	92.8 ± 15.5 [18]	89.8 ± 14.4 [7]	91.7 ± 15.2 [25]	0.060^(3)^
FEV_1_/FVC ratio	88.7 ± 12.5 [19]	91.4 ± 12.3 [7]	89.6 ± 12.4 [26]	0.045^(3)^

^(1)^The type of statin was not available for one patient.

^(2)^Chi square test.

^(3)^T test.

NA: Not Available. NS: Not Significant. FEV_1_: forced expiratory volume in 1 second; FVC: forced vital capacity; post-BD: post-bronchodilator. Square brackets indicate the number of missing values.

### Gene expression processing

**Fig 1:**
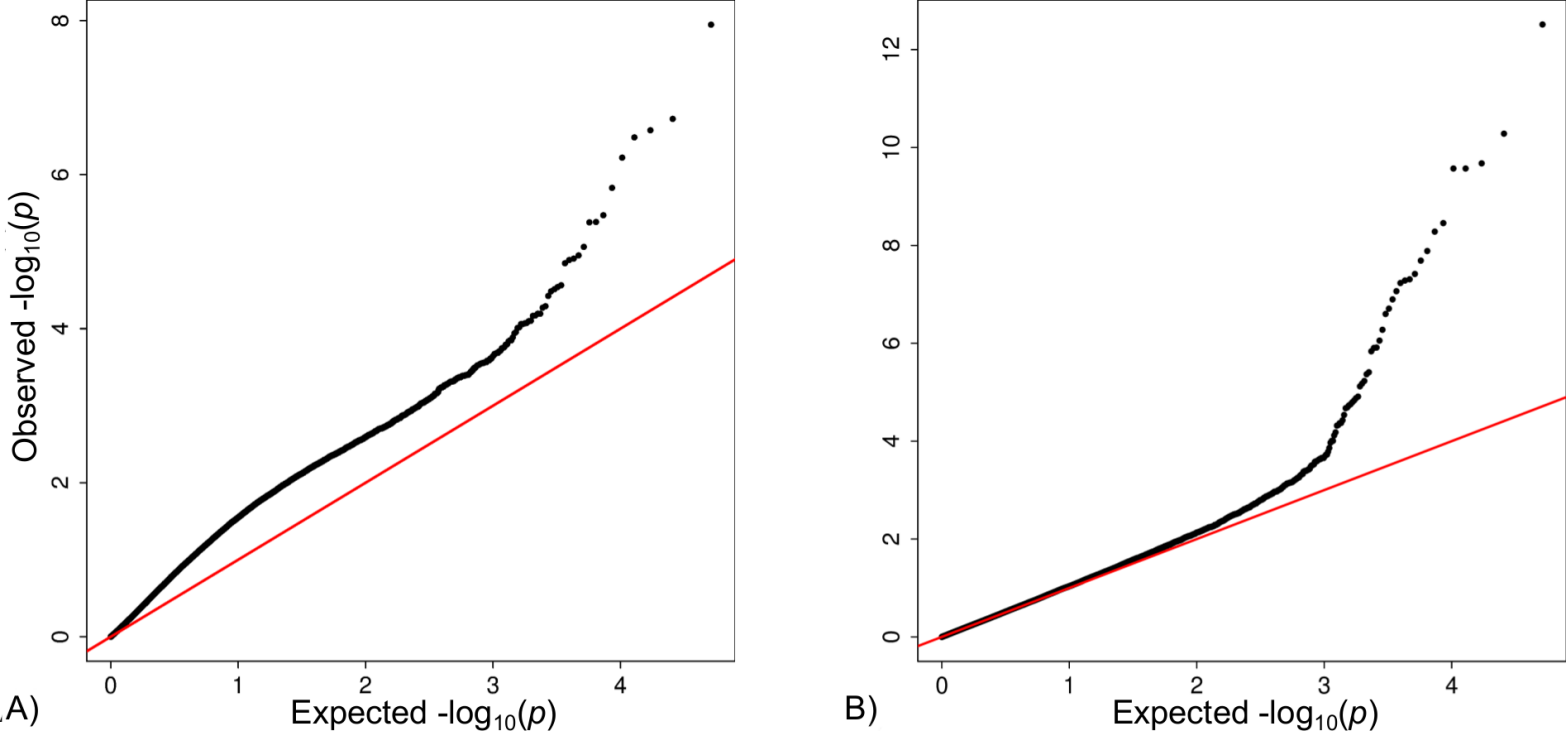
QQ-plots comparing adjustment methods. The QQ-plot is expected to follow the red line except for the extreme right (low P-values) end that may contain differentially expressed genes. A) QQ-plot of p-values obtained after linear regression of gene expression to test for genes differentially expressed between patients with and without statin therapy adjusted for age, gender and smoking status. B) QQ-plot of p-values obtained after adjusting gene expression for surrogate variables derived from SVA.

The QQ-plot showing the distribution of p-values testing all probe sets for association with the statin status is illustrated in Fig 1A. The distribution of observed p-values was inflated. Applying SVA yielded a well-distributed test statistic with no inflation. Fig 1B shows the results after adjustment for 34 surrogate variables detected by the SVA algorithm.

### Genes differentially expressed

**Fig 2:**
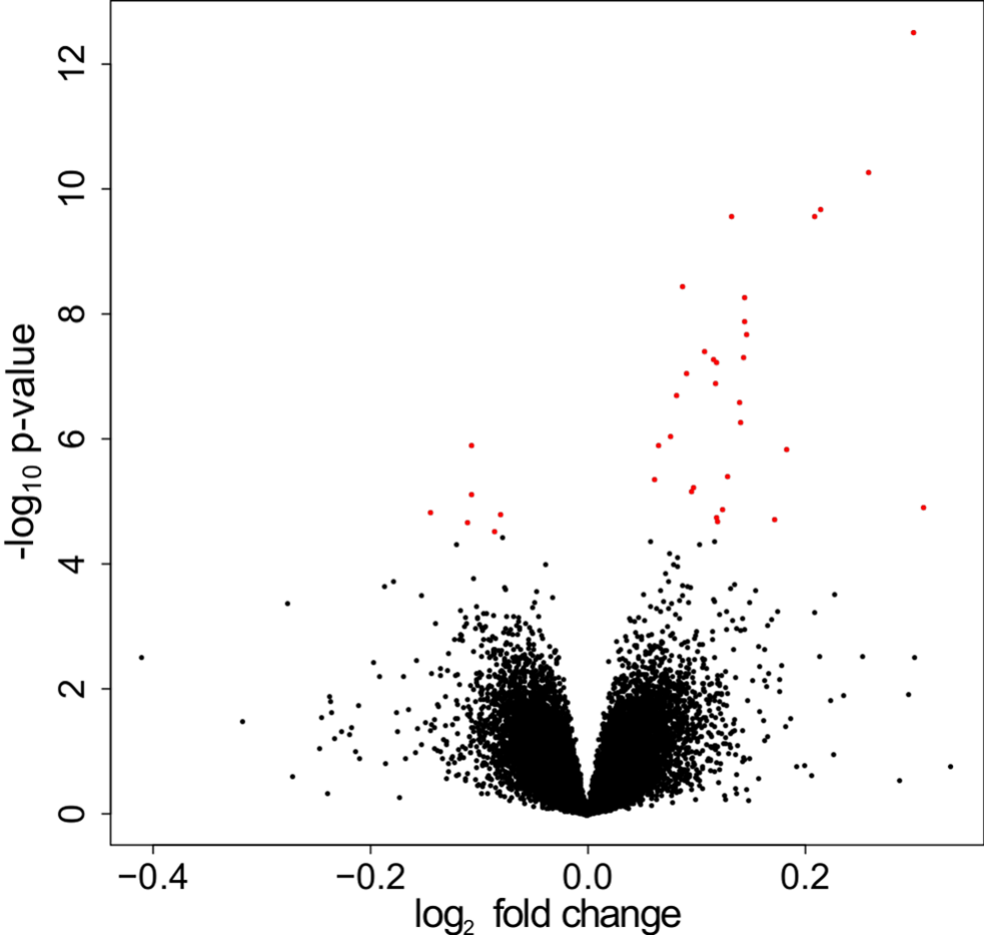
Volcano plot of SVA analysis p-values and fold changes. Presenting the impact of statin on gene expression in the lung obtained from the discovery set. Red dots correspond to genes claimed differentially expressed (FDR < 0.05).

In the analysis adjusted for surrogate variables, log 2 fold changes ranged from -0.41 to 0.34 (Fig 2). Twenty-one genes (34 probe sets) had a BH p-value below 0.05 and 12 genes (19 probe sets) had a p-value below the Bonferroni threshold of 9.55E-07 (α = 0.05) (Fig 2, Table 2). Eighteen genes were up-regulated and three were down-regulated with statin therapy.

**Table 2:** Genes (probe sets) differentially expressed between statin groups in the discovery and replication cohorts.

				Laval		Groningen	UBC	Meta-analysis	
Gene symbol	Gene name	log2FC	CI(±)	p value	BH	p value	p value	p value	BH
***HMGCS1***	3-hydroxy-3-methylglutaryl-CoA synthase 1	3.01E-01	7.81E-02	3.07E-13	1.61E-08	**1.70E-02**	3.00E-01	9.62E-13	4.95E-08
*HMGCS1*	3-hydroxy-3-methylglutaryl-CoA synthase 1	2.59E-01	7.53E-02	5.23E-11	1.37E-06	9.60E-02	5.06E-01	9.75E-10	8.37E-06
***TMEM97***	transmembrane protein 97	2.15E-01	6.48E-02	2.10E-10	2.80E-06	**1.38E-04**	7.18E-01	1.11E-11	2.85E-07
***HMGCS1***	3-hydroxy-3-methylglutaryl-CoA synthase 1	2.09E-01	6.33E-02	2.67E-10	2.80E-06	**3.48E-03**	2.10E-01	8.96E-11	1.15E-06
***TM7SF2***	transmembrane 7 superfamily member 2	1.33E-01	4.04E-02	2.68E-10	2.80E-06	**1.68E-03**	7.72E-02	1.77E-11	3.05E-07
***FDFT1***	farnesyl-diphosphate farnesyltransferase 1	8.77E-02	2.85E-02	3.49E-09	3.05E-05	**2.04E-03**	8.81E-01	2.26E-09	1.45E-05
***TMEM97***	transmembrane protein 97	1.45E-01	4.76E-02	5.22E-09	3.91E-05	**1.26E-03**	7.21E-01	1.75E-09	1.29E-05
***TMEM97***	transmembrane protein 97	1.45E-01	4.90E-02	1.30E-08	8.51E-05	**9.11E-04**	8.38E-01	3.45E-09	1.97E-05
***ACAT2***	acetyl-CoA acetyltransferase 2	1.47E-01	5.03E-02	2.04E-08	1.19E-04	**1.20E-03**	9.85E-01	7.78E-09	3.64E-05
***EBP***	emopamil binding protein	1.08E-01	3.79E-02	3.83E-08	2.01E-04	**3.78E-03**	1.58E-01	7.44E-09	3.64E-05
***ACAT2***	acetyl-CoA acetyltransferase 2	1.44E-01	5.10E-02	4.92E-08	2.28E-04	**1.15E-03**	7.68E-01	1.34E-08	5.76E-05
*MVD*	mevalonate (diphospho) decarboxylase	1.16E-01	4.12E-02	5.23E-08	2.28E-04	1.39E-01	3.28E-01	5.21E-07	1.58E-03
*SC4MOL*	methylsterol monooxygenase 1	1.20E-01	4.25E-02	5.87E-08	2.36E-04	6.44E-02	8.10E-01	6.51E-07	1.77E-03
***FDPS***	farnesyl diphosphate synthase	9.21E-02	3.32E-02	8.60E-08	3.22E-04	**6.39E-05**	4.41E-01	9.35E-10	8.37E-06
***HMGCR***	3-hydroxy-3-methylglutaryl-coenzyme A reductase	1.18E-01	4.32E-02	1.27E-07	4.43E-04	**2.93E-02**	7.05E-01	5.65E-07	1.62E-03
***FDFT1***	farnesyl-diphosphate farnesyltransferase 1	8.23E-02	3.05E-02	1.96E-07	6.42E-04	**1.17E-03**	8.46E-01	5.29E-08	2.10E-04
***SQLE***	squalene epoxidase	1.41E-01	5.26E-02	2.51E-07	7.72E-04	**1.13E-02**	3.19E-01	2.16E-07	6.95E-04
***SQLE***	squalene epoxidase	1.42E-01	5.45E-02	5.28E-07	1.54E-03	**5.98E-03**	1.36E-01	1.10E-07	4.04E-04
*AACS*	acetoacetyl-CoA synthetase	7.69E-02	3.02E-02	8.81E-07	2.43E-03	1.41E-01	2.94E-01	6.03E-06	1.24E-02
***FDFT1***	farnesyl-diphosphate farnesyltransferase 1	6.61E-02	2.64E-02	1.22E-06	3.09E-03	**1.10E-02**	8.51E-01	2.13E-06	4.99E-03
*CDK5RAP2*	CDK5 Regulatory Subunit Associated Protein 2	-1.06E-01	4.22E-02	1.24E-06	3.09E-03	1.30E-01	9.85E-01	2.22E-05	3.94E-02
*SC4MOL*	methylsterol monooxygenase 1	1.84E-01	7.39E-02	1.46E-06	3.48E-03	1.21E-01	4.08E-01	1.10E-05	2.03E-02
***DHCR7***	7-dehydrocholesterol reductase	1.30E-01	5.44E-02	3.92E-06	8.93E-03	**2.42E-03**	8.08E-01	1.49E-06	3.83E-03
***C14orf1***	Chromosome 14 Open Reading Frame 1	6.20E-02	2.61E-02	4.30E-06	9.39E-03	**4.51E-04**	3.76E-01	1.78E-07	6.10E-04
***HMGCR***	3-hydroxy-3-methylglutaryl-coenzyme A reductase	9.87E-02	4.22E-02	5.87E-06	1.23E-02	**3.49E-03**	8.57E-01	3.13E-06	6.71E-03
***HMGCR***	3-hydroxy-3-methylglutaryl-coenzyme A reductase	9.67E-02	4.16E-02	6.70E-06	1.35E-02	**6.72E-03**	9.59E-01	6.98E-06	1.38E-02
*FGFBP1*	Fibroblast Growth Factor Binding Protein 1	3.10E-01	1.37E-01	1.23E-05	2.30E-02	1.20E-01	2.93E-01	5.34E-05	8.59E-02
***INSIG1***	insulin induced gene 1	1.25E-01	5.58E-02	1.32E-05	2.39E-02	**6.06E-03**	1.14E-01	1.75E-06	4.28E-03
*ANAPC7*	Anaphase Promoting Complex Subunit 7	-7.94E-02	3.57E-02	1.60E-05	2.71E-02	9.08E-01	8.93E-01	9.81E-04	4.76E-01
***DHCR7***	7-dehydrocholesterol reductase	1.19E-01	5.40E-02	1.77E-05	2.89E-02	**1.85E-03**	4.42E-01	2.64E-06	5.90E-03
*ELOVL6*	ELOVL Fatty Acid Elongase 6	1.73E-01	7.83E-02	1.87E-05	2.97E-02	8.68E-01	2.97E-01	4.25E-04	3.36E-01
***INSIG1***	insulin induced gene 1	1.20E-01	5.47E-02	2.05E-05	3.16E-02	**1.51E-02**	1.79E-01	8.71E-06	1.66E-02
*EML1*	Echinoderm Microtubule Associated Protein Like 1	-1.09E-01	4.99E-02	2.11E-05	3.16E-02	1.17E-01	9.88E-01	2.38E-04	2.79E-01
*GINS3*	GINS Complex Subunit 3 (Psf3 homolog)	-8.46E-02	3.93E-02	2.92E-05	4.25E-02	9.31E-01	9.30E-01	1.71E-03	5.85E-01

CI is the confidence interval: the value to add and subtract to the log 2 fold change (Log2FC). BH is the Benjamini-Hochberg adjusted p-values. Genes in bold are replicated in at least one cohort (Groningen). Some genes are represented by more than one transcript.

### Replications sets

Whole-genome gene expression data were obtained from 445 patients in the Groningen set and 405 patients in the UBC set. After quality control filters, 341 and 282 patients had information on statin use and were of white European ancestry in Groningen and UBC, respectively. **Table 3** shows the demographic and clinical phenotypes for the two replication cohorts. Primary diagnosis in Groningen samples were lung cancer (n = 122), COPD (n = 69), cystic fibrosis (n = 44), and alpha-1 antitrypsin deficiency (n = 37). At UBC, the primary diagnosis was mainly lung cancer (n = 264). Twenty-six and twenty-four patients were taking statins in the Groningen and UBC cohorts, respectively. Of the 34 transcripts that were differentially expressed in the discovery cohort, 23 replicated in the Groningen cohort. Transcripts for *SC4MOL* (2 probe sets), *AACS*, *CDK5RAP2*, *FGFBP1*, *ANAPC7*, *ELOVL6*, *EML1*, *GINS3* and *MVD* as well as one out of three transcripts for HMGCS1 did not replicate. Overall, 12 unique genes up-regulated by statin treatment were replicated in the Groningen cohort, including *HMGCS1*, *TMEM97*, *TM7SF2*, *FDFT1*, *ACAT2*, *EBP*, *FDPS*, *HMGCR*, *SQLE*, *DHCR7*, *C14orf1*, and *INSIG1*. No transcript replicated in the UBC dataset.

**Table 3:** Clinical characteristics of the replication cohorts according to statin treatment.

	Groningen			UBC		
	Statins			Statins		
	**Non-users**	**Users**	**Total**	**Non-users**	**Users**	**Total**
	315	26	341	258	24	282
Gender						
Men	164	17	181	140	13	153
Women	151	9	160	118	11	129
Smoking status				(17 NAs)	(6 NAs)	(23 NAs)
Never	99	1	100	17	1	18
Former	165	19	184	138	12	150
Current	51	6	57	86	5	91
COPD	(102 NAs)	(2 NAs)	(104 NAs)	(48 NAs)	(5 NAs)	(53 NAs)
no	68	14	82	117	7	124
yes	145	10	155	93	12	105
Diabetes				(226 NAs)	(21 NAs)	(247 NAs)
no	295	19	314	21	1	22
yes	20	7	27	11	2	13
Age (mean ± SD)	50.34 ±50.4	65.19 ±8.87	51.47 ±15.65	62.84 ±11.44	68.88 ±6.53	63.35 ±11.22
BMI (mean ± SD, [NAs])	22.95 ±4.19, [27]	25.94 ±4.05, [3]	23.17 ±4.25, [30]	25.82 ±5.33, [26]	25.36 ±4.6, [5]	25.27 ±5.27, [31]
Pack year (mean ± SD, [NAs])	20.05 ±20.16, [26]	34.32 ±17.05, [4]	21.06 ±20.26, [30]	42.24 ±30.49, [26]	43.59 ±26.57, [3]	42.35 ±30.14, [29]
Primary diagnosis						
Lung cancer	100	22	122	241	23	264
Adenocarcinoma	24	12	36	74	10	84
Squamous cell carcinoma	46	5	51	75	8	83
Others	30	5	35	92	5	97
COPD	67	2	69	2	0	2
Cystic fibrosis	44	0	44	2	0	2
Alpha-1 antitrypsin deficiency	37	0	37	0	0	0
Others	67	2	69	13	1	14

NA: not available.

Considering the relatively small sample sizes of patients taking statins in the replication sets, we have also performed the analyses by combining the two cohorts. In this case, 20 out of 34 transcripts that were differentially expressed in the discovery cohort were replicated in the merged Groningen-UBC set. Results are shown in S3 Table. Genes validated by combining the two replication sets are the same 12 genes described above.

Finally, we performed a joint meta-analysis including the discovery and the two replication sets. This meta-analysis improved the significance of 12 of the up-regulated genes (Table 2). Twenty-nine transcripts corresponding to 16 genes were differentially expressed. In addition to the 12 genes replicated in at least one replication set and in the combined Groningen-UBC set, *MVD*, *SC4MOL*, *AACS*, and *CDK5RAP2* were also differently expressed. However, five genes differentially expressed in the discovery set were not significant in the meta-analysis including *FGFBP1*, *ANAPC7*, *ELOVL6*, *EML1* and *GINS3*.

### qPCR

**Fig 3:**
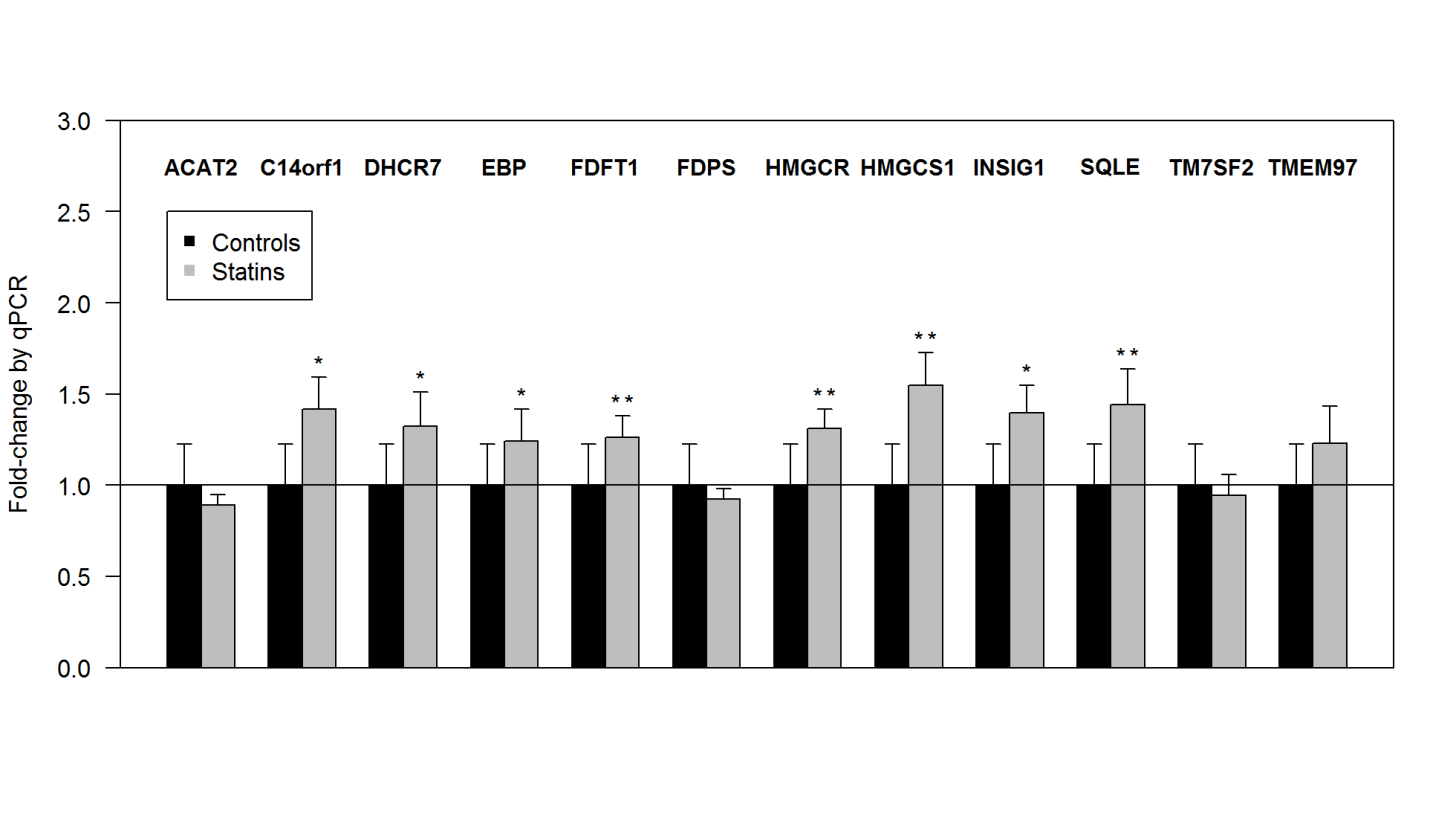
Validation of genes up-regulated by statin in lung tissues by qPCR. Patients taking (n = 20) and not taking (n = 20) statin were matched for gender, age and smoking status. Error bars are SE on a fold-change scale. *p < 0.05, **p < 0.01.

Twelve genes up-regulated by statin treatment and that were validated in at least one replication set were further validated by qPCR in 40 independent patients. Fig 3 shows gene expression between patients taking (n = 20) and not taking (n = 20) statin. Eight out of 12 genes were significantly up-regulated in statin users.

### Biological pathways

**Fig 4:**
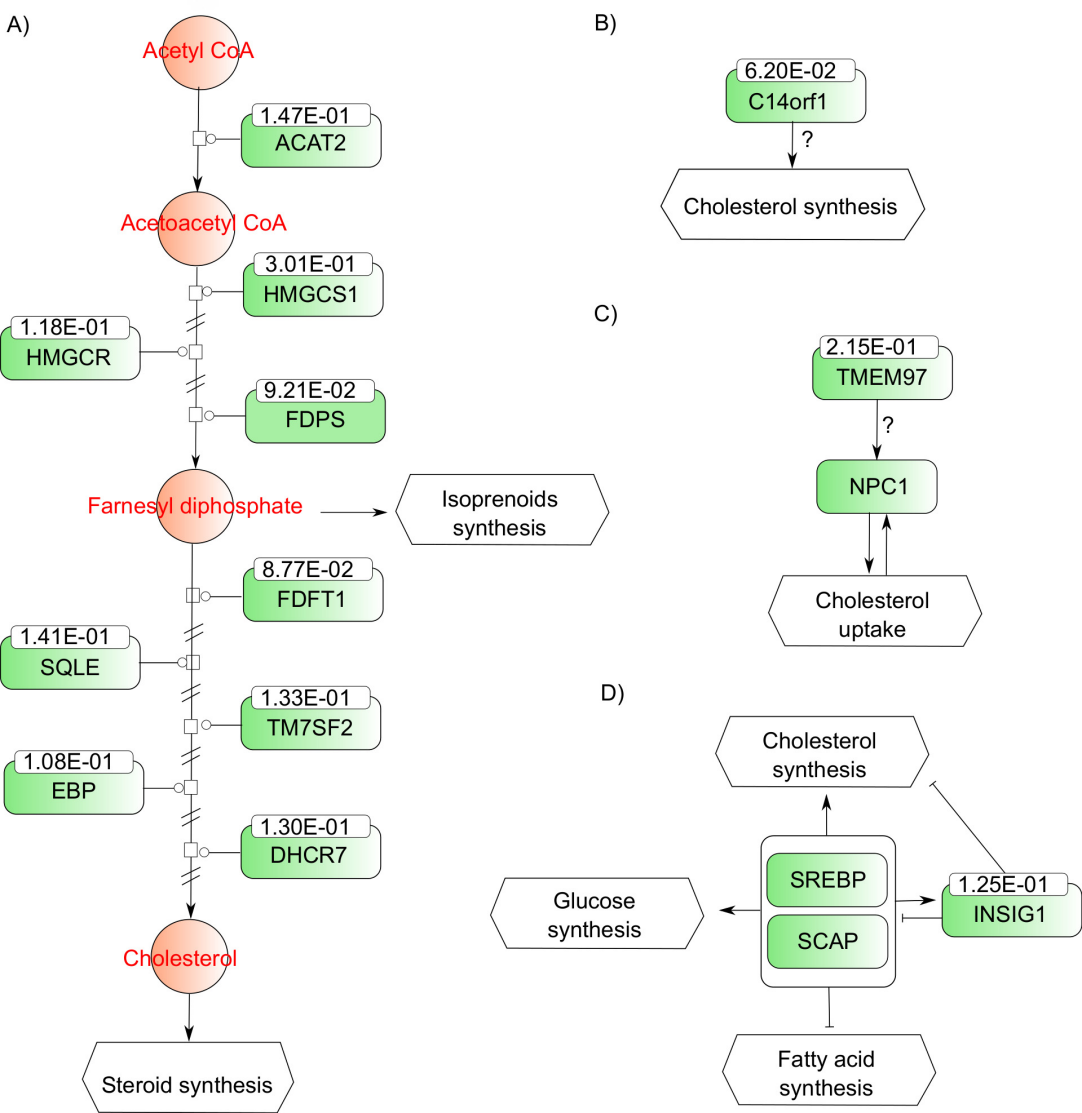
Biological pathways of genes detected as up-regulated by statins in lung tissue. Molecules and proteins are represented by orange circles and green rectangles, respectively. Squares on lines symbolize an enzymatic reaction. Lines ending with a circle designate the activity of an enzyme. Double slashes on line indicate missing step or information. A question mark indicates a hypothetical relation. Log 2 fold changes of differentially expressed genes are indicated on the top of rectangles. (A) Genes encoding enzymes and membrane proteins involved in cholesterol synthesis that are up-regulated by statins in lung tissues. (B) (C) and (D) illustrated the possible effects of C14orf1, INSIG1 and TMEM97 on cholesterol metabolism.

Out of the initial 52,378 probes sets, 36,650 were annotated with a gene name including 18,402 unique genes. GSEA was used on the 18,402 genes pre-ranked based on differential expression between patients with or without statins. Twenty-seven gene sets had a FDR q-value < 0.05 (Table 4). Three were from canonical pathway databases (i.e. REACTOME and KEGG). The REACTOME cholesterol biosynthesis pathway includes eight of the 12 genes up-regulated and validated in the lung of statin users namely *HMGCS1*, *TM7SF2*, *FDFT1*, *EBP*, *FDPS*, *HMGCR*, *SQLE*, and *DHCR7*. These are enzymes or membrane proteins of the endoplasmic reticulum implicated in the synthesis of cholesterol (S2 Fig). The KEGG steroid biosynthesis includes five genes up-regulated by statins namely *TM7SF2*, *FDFT1*, *EBP*, *SQLE*, and *DHCR7*. Finally the KEGG terpenoid backbone biosynthesis pathway includes four genes up-regulated by statins namely *HMGCS1*, *FDPS*, *HMGCR*, and *ACAT2*. All genes up-regulated by statins in these two KEGG pathways, except *ACAT2*, were also found in the REACTOME cholesterol biosynthesis pathway. Fig 4A summarizes genes up-regulated by statins in the lung and implicated in the synthesis of cholesterol.

**Table 4:** Summary of top GSEA gene sets with a FDR q-value < 0.05.

Name	Nominal p-val	FDR q-val	FWER p-val
HORTON_SREBF_TARGETS	<2.16E-03	<3.47E-04	<3.00E-03
SCHMIDT_POR_TARGETS_IN_LIMB_BUD_UP	<2.16E-03	<3.47E-04	<3.00E-03
**REACTOME_CHOLESTEROL_BIOSYNTHESIS**	<2.16E-03	<3.47E-04	<3.00E-03
MODULE_432	<2.16E-03	<3.47E-04	<3.00E-03
**KEGG_STEROID_BIOSYNTHESIS**	<2.16E-03	<3.47E-04	<3.00E-03
**KEGG_TERPENOID_BACKBONE_BIOSYNTHESIS**	<2.16E-03	<3.47E-04	<3.00E-03
CSR_LATE_UP.V1_DN	<2.16E-03	<3.47E-04	<3.00E-03
WENG_POR_TARGETS_GLOBAL_UP	<2.16E-03	<3.47E-04	<3.00E-03
WILCOX_PRESPONSE_TO_ROGESTERONE_UP	<2.16E-03	<3.47E-04	<3.00E-03
PODAR_RESPONSE_TO_ADAPHOSTIN_DN	<2.16E-03	3.47E-04	3.00E-03
JI_RESPONSE_TO_FSH_UP	<2.16E-03	5.27E-04	5.00E-03
WENG_POR_TARGETS_LIVER_UP	<2.16E-03	1.45E-03	1.50E-02
ZWANG_EGF_PERSISTENTLY_UP	<2.16E-03	1.34E-03	1.50E-02
GUO_TARGETS_OF_IRS1_AND_IRS2	<2.16E-03	6.69E-03	7.60E-02
MTOR_UP.V1_UP	<2.16E-03	8.10E-03	9.90E-02
STEROID_BIOSYNTHETIC_PROCESS	<2.16E-03	7.67E-03	1.00E-01
MITOCHONDRION_ORGANIZATION_AND_BIOGENESIS	<2.16E-03	9.00E-03	1.23E-01
GSE24634_IL4_VS_CTRL_TREATED_NAIVE_CD4_TCELL_DAY5_UP	<2.16E-03	1.27E-02	1.76E-01
GNF2_IL2RB	<2.16E-03	2.34E-02	3.05E-01
CHR1Q31	<2.16E-03	2.46E-02	3.34E-01
MITOCHONDRIAL_TRANSPORT	<2.16E-03	2.60E-02	3.65E-01
CAFFAREL_RESPONSE_TO_THC_DN	<2.16E-03	2.62E-02	3.82E-01
LE_EGR2_TARGETS_DN	<2.16E-03	2.90E-02	4.30E-01
GNF2_PTPN4	2.16E-03	4.30E-02	5.73E-01
ZHANG_GATA6_TARGETS_DN	<2.16E-03	4.73E-02	6.25E-01
GNF2_CD7	<2.16E-03	4.59E-02	6.28E-01
BURTON_ADIPOGENESIS_10	<2.16E-03	4.95E-02	6.68E-01

FDR: False Discovery Rate; FWER: FamilyWise Error Rate.

Gene sets from canonical pathway databases are shown in bold.

### Sub-group analysis excluding patients with severe-to-very-severe COPD

A total of 385 patients without severe COPD (GOLD stages 3 and 4) were selected in the discovery cohort including 134 statin users. The Groningen and UBC replication cohorts comprised 244 and 120 COPD patients without severe or very severe disease including 18 and 19 statin users, respectively. Applying linear regression adjusted for age, gender and smoking status as well as surrogate variables from SVA in the discovery cohort yielded 33 transcripts differentially expressed (BH p-value < 0.05) in patients taking or not statins, which corresponded to 21 genes (S4 Table). Three genes were down-regulated and 18 were up-regulated in statin users and all overlapped with genes found in the main analysis. Seventeen transcripts corresponding to 9 genes were replicated in Groningen (p-value < 0.05), but none in UBC. Replicated genes in Groningen include *HMGCS1*, *TMEM97*, *ACAT2*, *HMGCR*, *SC4MOL*, *FDPS*, *SQLE*, *CDK5RAP2*, and *INSIG1*. In the joint meta-analysis, 27 transcripts corresponding to 15 genes were differentially expressed, which include the nine genes plus *TM7SF2*, *FDFT1*, *MVD*, *EBP*, *C14orf1*, and *FGFBP1*. Biological pathways were similar to those found in the main analysis.

## Discussion

The goal of this study was to identify genes and biological pathways that are modulated by statin treatment in human lung that may explain the improved respiratory health and survival observed in patients with lung diseases taking this class of lipid-lowering drug. Statins are known to alter cholesterol synthesis. Twenty-one genes were differentially expressed by statin treatment in the lung. Twelve up-regulated genes replicated in one independent set including genes encoding enzymes and membrane proteins involved in cholesterol biosynthesis. Eight of these genes were further validated by qPCR in an independent set of lung specimens. Canonical biological pathways altered by statins in the lung include cholesterol, steroid, and terpenoid backbone biosynthesis. No genes encoding proteases, growth factors, pro-fibrotic or pro-inflammatory mediators were identified that may have explained the pleiotropic effects of statins in the lung. The sub-analysis of patients with no severe COPD supports these results.

All 12 genes up-regulated and replicated by statins in the lung were also found to be up-regulated in primary human hepatocytes treated with either atorvastatin or rosuvastatin [38], which provides an external validation of our results. Eight out of the 12 genes were part of the REACTOME cholesterol biosynthesis pathway including *HMGCS1*, *TM7SF2*, *FDFT1*, *EBP*, *FDPS*, *HMGCR*, *SQLE*, and *DHCR7*. *MVD*, belonging to this pathway, was up-regulated by statins in the discovery set, but was not replicated (S2 Fig). *ACAT2* was also up-regulated by statins in the lung. This enzyme is responsible to convert acetyl-CoA to acetoacetyl-CoA, which is the precursor feeding the cholesterol biosynthesis pathway (Fig 4A). The current study thus confirmed that cardio-protective doses of statin reached the lung and mediated an anti-lipidemic action.

Three other genes were found up-regulated in the lung of statin users namely *INSIG*, *TMEM97*, and *C14orf1*. INSIG1 is an endoplasmic reticulum membrane protein that binds SCAP (SREBP cleavage-activating protein) and HMG CoA reductase. INSIG1 mediated sterol-induced ubiquitination and ER-associated degradation of reductase, and thus plays a critical role in regulating cholesterol concentration in cells [52]. TMEM97 was identified as a functional regulator of cellular cholesterol homeostatis [53]. Finally, *C14orf1* (also known as *ERG28*) was also shown to be involved in sterol biosynthesis [54]. *TMEM97*, *INSIG1*, and *C14orf1* are thus in line with the cholesterogenic action of statins in the lung.

A recent prospective randomized controlled trial of patients with moderate to severe COPD showed that simvastatin had no beneficial effect on the number of exacerbations or the time to a first exacerbation [28]. As stated by the authors, the beneficial effect of statins may be restricted to patients with less severe COPD. Moreover, patients with cardiovascular disease were excluded in that prospective trial. Following this publication, a retrospective study found that statin use was associated with reduced odds of exacerbations in individuals with COPD, but not in patients with severe COPD without cardiovascular comorbidity [55]. In the current study, keeping or removing patients with severe COPD had a minimal effect on the impact of statins on gene expression in the lung.

This study has limitations. The lung is composed of different cell types that vary in proportion in different conditions and individuals. Gene expression levels are sensitive to the abundance of different cell types including lung specific cells (e.g. pneumocytes) and immune specific cells (e.g. alveolar macrophages). In addition, the types of statin were not considered. Statin classes differ in their biological effects due to specific characteristics such as lipophilicity responsible for their absorption, metabolism and excretion. In this study, most patients were taking lipophilic statins which are active in the liver and extrahepatic organs whereas hydrophilic statins are more selective for the liver [56]. In addition, no information is available on statin dosage. Differences in statin types and dosage may explain the lack of replication in the UBC replication set. However, the lack of power (only 24 statin users) may also contribute to this observation. Furthermore, gene expression is only one aspect of the molecular alterations induced by statins in the lung. These results will need to be confirmed by other studies looking for other biological dimensions such as proteomic and epigenetic changes.

## Conclusion

Beyond their antilipidemic action, statins are known to have many pharmacological effects such as antioxidant, antithrombotic, antiarrhythmic, antifibrotic, anticancer, antiapoptotic, antiproliferative and antiinflammatory. In this study, we analyzed the impact of statins on gene expression in the lung in order to elucidate in humans the molecular mechanisms underpinning the clinical benefits of statins in chronic lung diseases. Results indicate that statins up-regulate genes encoding enzymes and membrane proteins involved in cholesterol synthesis. Our study design was promising to pinpoint genes and/or molecular pathways altered in the lung of statin users and reveal the specific pleiotropic effect(s) of statins that is taking place in the lung. However, no genes were altered beyond those implicated in the anti-lipidemic action of statins. This genome-wide gene expression study is thus not supporting direct pleiotropic effects of statin in lung tissues. Based on these results and bearing in mind the aforementioned limitations, the improved health outcomes and survival observed in statin users with chronic lung diseases may be more likely mediated by the reduction of systemic inflammation and/or the indirect effects of statins on cardiovascular comorbidities. The next steps will require gene expression in specific lung cell types, *in silico* deconvolution approaches [57], and further studies using different classes of statins with known dosages.

## Supporting Information

S1 Fig Overview of the analysis framework. Global input and output are represented in red. Actions are represented in blue with more details in black.
(DOCX)

S2 Fig The REACTOME cholesterol biosynthesis pathway. Genes circled in red were found up-regulated in the lung of statin users. The gene circled in blue was up-regulated by statins in the discovery set, but not validated in the replication sets.
(DOCX)

S1 Table Clinical characteristics of patients used in the qPCR experiment. (DOCX)

S2 Table Primers used for the quantitative real-time PCR (qPCR). (DOCX)

S3 Table Validation of genes (probe sets) differentially expressed between statin groups in the combined Groningen-UBC set. (DOCX)

S4 Table Genes (probe sets) differentially expressed between statin groups in the discovery and replication cohorts excluding patients with severe COPD. (DOCX)
