# Supplementary material for: Impact of Statins on Gene Expression in Human Lung Tissues
Source: PLoS One. 2015 Nov 4;10(11):e0142037. doi: 10.1371/journal.pone.0142037 (PMC4633125; doi:10.1371/journal.pone.0142037)
Supplement: S1 Fig — Global input and output are represented in red. Actions are represented in blue with more details in black. (DOCX) [file pone.0142037.s001.docx]

**
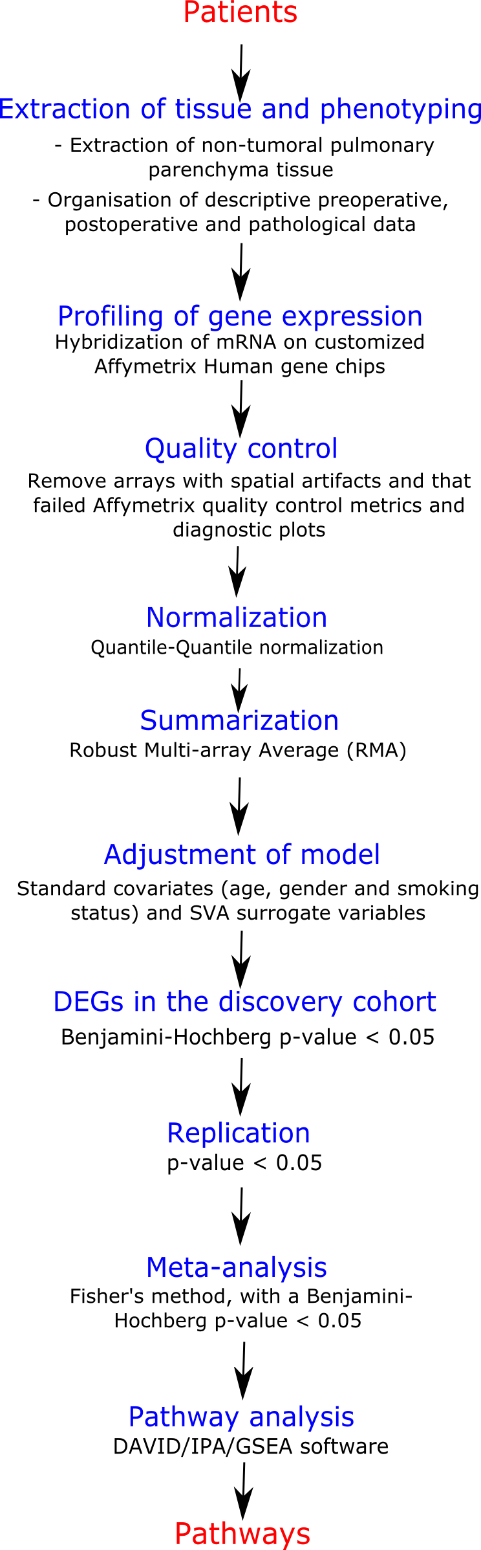
**

**S1 Fig**. Overview of the analysis framework. Global input and output are represented in red. Actions are represented in blue with more details in black.
