# Supplementary material for: Impact of Statins on Gene Expression in Human Lung Tissues
Source: PLoS One. 2015 Nov 4;10(11):e0142037. doi: 10.1371/journal.pone.0142037 (PMC4633125; doi:10.1371/journal.pone.0142037)
Supplement: S2 Fig — Genes circled in red were found up-regulated in the lung of statin users. The gene circled in blue was up-regulated by statins in the discovery set, but not validated in the replication sets. (DOCX) [file pone.0142037.s002.docx]

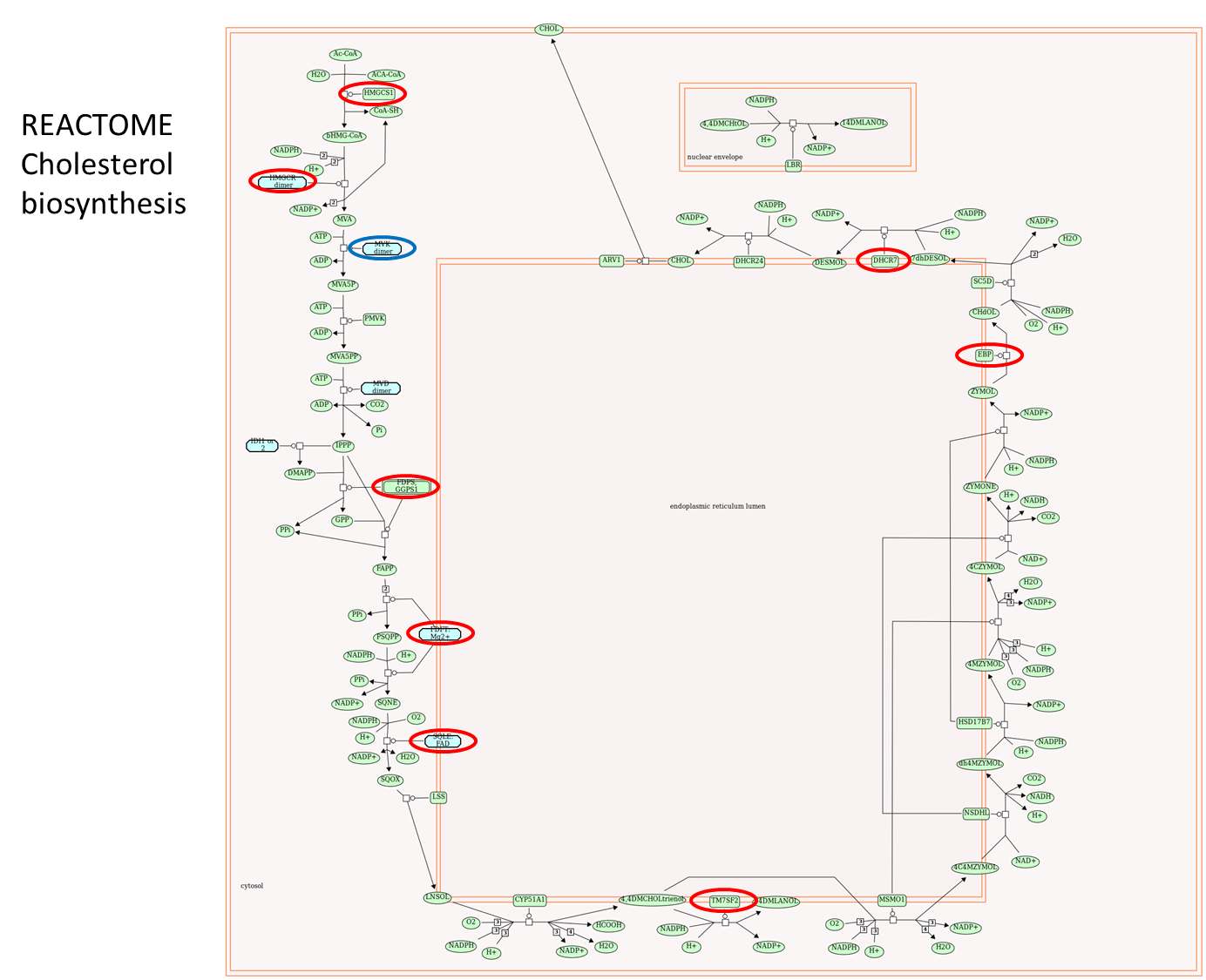


**S2 Fig**. The REACTOME cholesterol biosynthesis pathway. Genes circled in red were found up-regulated in the lung of statin users. The gene circled in blue was up-regulated by statins in the discovery set, but not validated in the replication sets.
