# Supplementary material for: Impact of Statins on Gene Expression in Human Lung Tissues
Source: PLoS One. 2015 Nov 4;10(11):e0142037. doi: 10.1371/journal.pone.0142037 (PMC4633125; doi:10.1371/journal.pone.0142037)
Supplement: S1 Table — (DOCX) [file pone.0142037.s003.docx]

**S1 Table**. Clinical characteristics of patients used in the qPCR experiment

|  | **Statin (n = 20)** | **Non-statin (n = 20)** |
| --- | --- | --- |
| Statin types |  |  |
| Atorvastatin | 9 | - |
| Simvastatin | 1 | - |
| Rosuvastatin | 10 | - |
| Gender |  |  |
| Male | 10 | 10 |
| Female | 10 | 10 |
| Smoking status |  |  |
| Never | 2 | 2 |
| Former | 11 | 11 |
| Current | 7 | 7 |
| Age (mean ± SD) | 66.20 ± 6.30 | 66.26 ± 7.12 |
| Pack-years (mean ± SD) | 46.58 ± 14.15 | 45.5 ± 12.41 |
| Lung cancer histology |  |  |
| Adenocarcinoma | 9 | 9 |
| Carcinoid | 1 | 1 |
| Squamous cell carcinoma | 10 | 10 |
