# Supplementary material for: Impact of Statins on Gene Expression in Human Lung Tissues
Source: PLoS One. 2015 Nov 4;10(11):e0142037. doi: 10.1371/journal.pone.0142037 (PMC4633125; doi:10.1371/journal.pone.0142037)
Supplement: S2 Table — (DOCX) [file pone.0142037.s004.docx]

**S2 Table**. Primers used for the quantitative real-time PCR (qPCR).

| **Gene** | **Forward oligo 5’-3’** | **Reverse oligo 5’-3’** | **Final oligo conc (µM)** | **Amplicon size** | **Annealing Tm** |
| --- | --- | --- | --- | --- | --- |
| HMGCS1 | CTTTGGATGAAGGAGTAGGACTTGTGC | GCTGCTTCAGGTTCTGCTGC | 0.5 | 112 | 61 °C |
| TMEM97 | CTTTCCCATTGCAACGTATGCCTTC | AGGTCTTTGTCCCTTGAAACCACTG | 0.5 | 163 | 60 °C |
| TM7SF2 | TCATCATGGCTCTGGCTTGGTC | TACTTCTGCAGGCACTGCCG | 0.5 | 130 | Touchdown |
| FDFT1 | GGAAAGGGCAAGCAGTGACC | GGATGGTGGAGATGATCTGCCTT | 0.5 | 150 | 60 °C |
| ACAT2 | CCCAGAGAAGGTCAATATTGAAGGAGG | CTCCAGTGTGTGTAACAGGGTCAC | 0.5 | 100 | 60 °C |
| EBP | CTTGTGGTCTCTGTGGGCCA | AGCACAAGGACTCCAGGCAG | 0.5 | 161 | Touchdown |
| FDPS | GCTGGTGGTTCAGTGTCTGC | GCAAGAACACTGCTGGCAGATC | 0.5 | 140 | 59 °C |
| HMGCR | AAGCCTGTTTGCAGATGCTAGG | GATGTCCTGCTGCCAATGCT | 0.5 | 141 | 60 °C |
| SQLE | TGGGCTGCTTTCTGTATTGTCTCC | CACCACTACTGAGAAGGGCTCG | 0.5 | 137 | 60 °C |
| DHCR7 | CTGTGTCTGGCTGCCTTATCTTTACAC | GAAGATGTAGTAGCCCACCAGGC | 0.5 | 124 | 60 °C |
| C14orf1 | CTGATGGTGGCAAGTTTCTCCATCC | GAGACGACGAAGGAAGAAGATGGC | 0.5 | 160 | 59 °C |
| INSIG1 | TGGGGATCACCATAGCTTTTCTAGCTAC | GTCCTATGTTCCCCACCGTGAC | 0.5 | 153 | 60 °C |
| *GAPDH | ATGTTCGTCATGGGTGTGAA | GGTGCTAAGCAGTTGGTGGT | 0.5 | 89 | 59 °C |
| *ACTB | ACTTCGAGCAAGAGATGGCCAC | GGACTCCATGCCCAGGAAGG | 0.5 | 149 | 59 °C |
| *B2M | GAGTGCTGTCTCCATGTTTGATGT | AAGTTGCCAGCCCTCCTAGAG | 0.5 | 71 | 60 °C |

*Reference genes.
