# Supplementary material for: Impact of Statins on Gene Expression in Human Lung Tissues
Source: PLoS One. 2015 Nov 4;10(11):e0142037. doi: 10.1371/journal.pone.0142037 (PMC4633125; doi:10.1371/journal.pone.0142037)
Supplement: S3 Table — (DOCX) [file pone.0142037.s005.docx]

**S3 Table**. Validation of genes (probe sets) differentially expressed between statin groups in the combined Groningen-UBC set.

|  |  |  |  | **Laval** |  | **Groningen - UBC** |
| --- | --- | --- | --- | --- | --- | --- |
| **Gene symbol** | **Gene name** | **log2FC** | **CI(±)** | **p value** | **BH** | **p value** |
| ***HMGCS1*** | 3-hydroxy-3-methylglutaryl-CoA synthase 1 | 3.01E-01 | 7.81E-02 | 3.07E-13 | 1.61E-08 | **1.45E-03** |
| ***HMGCS1*** | 3-hydroxy-3-methylglutaryl-CoA synthase 1 | 2.59E-01 | 7.53E-02 | 5.23E-11 | 1.37E-06 | **3.51E-02** |
| ***TMEM97*** | transmembrane protein 97 | 2.15E-01 | 6.48E-02 | 2.10E-10 | 2.80E-06 | **2.58E-04** |
| ***HMGCS1*** | 3-hydroxy-3-methylglutaryl-CoA synthase 1 | 2.09E-01 | 6.33E-02 | 2.67E-10 | 2.80E-06 | **2.22E-03** |
| ***TM7SF2*** | transmembrane 7 superfamily member 2 | 1.33E-01 | 4.04E-02 | 2.68E-10 | 2.80E-06 | **1.25E-04** |
| ***FDFT1*** | farnesyl-diphosphate farnesyltransferase 1 | 8.77E-02 | 2.85E-02 | 3.49E-09 | 3.05E-05 | **2.60E-02** |
| ***TMEM97*** | transmembrane protein 97 | 1.45E-01 | 4.76E-02 | 5.22E-09 | 3.91E-05 | **1.61E-03** |
| ***TMEM97*** | transmembrane protein 97 | 1.45E-01 | 4.90E-02 | 1.30E-08 | 8.51E-05 | **1.60E-03** |
| ***ACAT2*** | acetyl-CoA acetyltransferase 2 | 1.47E-01 | 5.03E-02 | 2.04E-08 | 1.19E-04 | **7.90E-03** |
| ***EBP*** | emopamil binding protein | 1.08E-01 | 3.79E-02 | 3.83E-08 | 2.01E-04 | **3.52E-04** |
| ***ACAT2*** | acetyl-CoA acetyltransferase 2 | 1.44E-01 | 5.10E-02 | 4.92E-08 | 2.28E-04 | **3.16E-03** |
| *MVD* | mevalonate (diphospho) decarboxylase | 1.16E-01 | 4.12E-02 | 5.23E-08 | 2.28E-04 | 6.20E-02 |
| *SC4MOL* | methylsterol monooxygenase 1 | 1.20E-01 | 4.25E-02 | 5.87E-08 | 2.36E-04 | 1.40E-01 |
| ***FDPS*** | farnesyl diphosphate synthase | 9.21E-02 | 3.32E-02 | 8.60E-08 | 3.22E-04 | **1.11E-04** |
| ***HMGCR*** | 3-hydroxy-3-methylglutaryl-coenzyme A reductase | 1.18E-01 | 4.32E-02 | 1.27E-07 | 4.43E-04 | 6.97E-02 |
| ***FDFT1*** | farnesyl-diphosphate farnesyltransferase 1 | 8.23E-02 | 3.05E-02 | 1.96E-07 | 6.42E-04 | **2.42E-02** |
| ***SQLE*** | squalene epoxidase | 1.41E-01 | 5.26E-02 | 2.51E-07 | 7.72E-04 | **6.21E-03** |
| ***SQLE*** | squalene epoxidase | 1.42E-01 | 5.45E-02 | 5.28E-07 | 1.54E-03 | **2.69E-03** |
| *AACS* | acetoacetyl-CoA synthetase | 7.69E-02 | 3.02E-02 | 8.81E-07 | 2.43E-03 | 1.15E-01 |
| ***FDFT1*** | farnesyl-diphosphate farnesyltransferase 1 | 6.61E-02 | 2.64E-02 | 1.22E-06 | 3.09E-03 | 1.19E-01 |
| *CDK5RAP2* | CDK5 Regulatory Subunit Associated Protein 2 | -1.06E-01 | 4.22E-02 | 1.24E-06 | 3.09E-03 | 2.18E-01 |
| *SC4MOL* | methylsterol monooxygenase 1 | 1.84E-01 | 7.39E-02 | 1.46E-06 | 3.48E-03 | 3.40E-01 |
| ***DHCR7*** | 7-dehydrocholesterol reductase | 1.30E-01 | 5.44E-02 | 3.92E-06 | 8.93E-03 | **8.44E-03** |
| ***C14orf1*** | Chromosome 14 Open Reading Frame 1 | 6.20E-02 | 2.61E-02 | 4.30E-06 | 9.39E-03 | **1.11E-03** |
| ***HMGCR*** | 3-hydroxy-3-methylglutaryl-coenzyme A reductase | 9.87E-02 | 4.22E-02 | 5.87E-06 | 1.23E-02 | **1.25E-02** |
| ***HMGCR*** | 3-hydroxy-3-methylglutaryl-coenzyme A reductase | 9.67E-02 | 4.16E-02 | 6.70E-06 | 1.35E-02 | **2.87E-02** |
| *FGFBP1* | Fibroblast Growth Factor Binding Protein 1 | 3.10E-01 | 1.37E-01 | 1.23E-05 | 2.30E-02 | 5.37E-02 |
| ***INSIG1*** | insulin induced gene 1 | 1.25E-01 | 5.58E-02 | 1.32E-05 | 2.39E-02 | 1.49E-01 |
| *ANAPC7* | Anaphase Promoting Complex Subunit 7 | -7.94E-02 | 3.57E-02 | 1.60E-05 | 2.71E-02 | 5.36E-01 |
| ***DHCR7*** | 7-dehydrocholesterol reductase | 1.19E-01 | 5.40E-02 | 1.77E-05 | 2.89E-02 | **3.60E-03** |
| *ELOVL6* | ELOVL Fatty Acid Elongase 6 | 1.73E-01 | 7.83E-02 | 1.87E-05 | 2.97E-02 | 4.48E-01 |
| ***INSIG1*** | insulin induced gene 1 | 1.20E-01 | 5.47E-02 | 2.05E-05 | 3.16E-02 | 2.18E-01 |
| *EML1* | Echinoderm Microtubule Associated Protein Like 1 | -1.09E-01 | 4.99E-02 | 2.11E-05 | 3.16E-02 | 4.37E-01 |
| *GINS3* | GINS Complex Subunit 3 (Psf3 homolog) | -8.46E-02 | 3.93E-02 | 2.92E-05 | 4.25E-02 | 9.54E-01 |

CI is the confidence interval: the value to add and subtract to the log 2 fold change (Log2FC). BH is the Benjamini-Hochberg adjusted p-values. Genes in bold are replicated in the combined Groningen-UBC set. Some genes are represented by more than one transcript.
