# Supplementary material for: Impact of Statins on Gene Expression in Human Lung Tissues
Source: PLoS One. 2015 Nov 4;10(11):e0142037. doi: 10.1371/journal.pone.0142037 (PMC4633125; doi:10.1371/journal.pone.0142037)
Supplement: S4 Table — (DOCX) [file pone.0142037.s006.docx]

**S4 Table**. Genes (probe sets) differentially expressed between statin groups in the discovery and replication cohorts excluding patients with severe COPD.

|  |  | **Laval** | | | | **Groningen** | **UBC** | **Meta-analysis** | |
| --- | --- | --- | --- | --- | --- | --- | --- | --- | --- |
| **Gene symbol** | **Gene name** | **log2FC** | **CI(±)** | **p value** | **BH** | **p value** | **p value** | **p value** | **BH** |
| ***HMGCS1*** | 3-hydroxy-3-methylglutaryl-CoA synthase 1 | 3.08E-01 | 8.04E-02 | 4.49E-13 | 2.35E-08 | **1.26E-03** | 5.24E-01 | 2.00E-13 | 1.03E-08 |
| ***HMGCS1*** | 3-hydroxy-3-methylglutaryl-CoA synthase 1 | 2.84E-01 | 7.63E-02 | 1.78E-12 | 4.67E-08 | **5.82E-03** | 8.04E-01 | 4.66E-12 | 1.20E-07 |
| *HMGCS1* | 3-hydroxy-3-methylglutaryl-CoA synthase 1 | 2.21E-01 | 6.52E-02 | 1.00E-10 | 1.75E-06 | 5.03E-02 | 2.51E-01 | 5.12E-10 | 8.80E-06 |
| ***TMEM97*** | transmembrane protein 97 | 2.21E-01 | 6.71E-02 | 3.26E-10 | 4.27E-06 | **2.44E-02** | 9.20E-01 | 2.60E-09 | 2.68E-05 |
| *TM7SF2* | transmembrane 7 superfamily member 2 | 1.33E-01 | 4.11E-02 | 5.72E-10 | 5.99E-06 | 1.51E-01 | 7.69E-02 | 2.37E-09 | 2.68E-05 |
| *FDFT1* | farnesyl-diphosphate farnesyltransferase 1 | 9.09E-02 | 2.93E-02 | 2.68E-09 | 2.34E-05 | 1.78E-01 | 8.72E-01 | 1.06E-07 | 4.56E-04 |
| ***TMEM97*** | transmembrane protein 97 | 1.52E-01 | 4.96E-02 | 4.64E-09 | 3.47E-05 | **3.89E-02** | 8.06E-01 | 4.08E-08 | 2.65E-04 |
| ***TMEM97*** | transmembrane protein 97 | 1.52E-01 | 5.10E-02 | 1.16E-08 | 7.56E-05 | **2.07E-02** | 9.39E-01 | 6.07E-08 | 3.40E-04 |
| *ACAT2* | acetyl-CoA acetyltransferase 2 | 1.52E-01 | 5.16E-02 | 1.64E-08 | 9.54E-05 | 1.08E-02 | 8.27E-01 | 4.09E-08 | 2.65E-04 |
| ***ACAT2*** | acetyl-CoA acetyltransferase 2 | 1.52E-01 | 5.20E-02 | 2.03E-08 | 9.93E-05 | **7.40E-03** | 9.78E-01 | 4.12E-08 | 2.65E-04 |
| *MVD* | mevalonate (diphospho) decarboxylase | 1.23E-01 | 4.20E-02 | 2.09E-08 | 9.93E-05 | 9.50E-01 | 3.65E-01 | 1.41E-06 | 3.30E-03 |
| *EBP* | emopamil binding protein | 1.10E-01 | 3.90E-02 | 6.32E-08 | 2.76E-04 | 2.32E-01 | 2.53E-01 | 7.75E-07 | 2.35E-03 |
| ***HMGCR*** | 3-hydroxy-3-methylglutaryl-coenzyme A reductase | 1.23E-01 | 4.41E-02 | 7.54E-08 | 3.04E-04 | **5.99E-03** | 7.42E-01 | 8.74E-08 | 4.09E-04 |
| *FDFT1* | farnesyl-diphosphate farnesyltransferase 1 | 7.45E-02 | 2.68E-02 | 8.64E-08 | 3.06E-04 | 1.35E-01 | 5.71E-01 | 1.31E-06 | 3.22E-03 |
| ***SC4MOL*** | methylsterol monooxygenase 1 | 1.23E-01 | 4.44E-02 | 8.86E-08 | 3.06E-04 | **4.82E-02** | 9.35E-01 | 8.29E-07 | 2.37E-03 |
| *FDFT1* | farnesyl-diphosphate farnesyltransferase 1 | 8.70E-02 | 3.14E-02 | 9.36E-08 | 3.06E-04 | 1.16E-01 | 7.45E-01 | 1.56E-06 | 3.50E-03 |
| ***FDPS*** | farnesyl diphosphate synthase | 9.09E-02 | 3.36E-02 | 1.83E-07 | 5.63E-04 | **3.60E-02** | 4.44E-01 | 6.25E-07 | 2.01E-03 |
| ***SQLE*** | squalene epoxidase | 1.45E-01 | 5.37E-02 | 2.00E-07 | 5.83E-04 | **1.28E-02** | 2.95E-01 | 1.83E-07 | 7.25E-04 |
| ***SQLE*** | squalene epoxidase | 1.48E-01 | 5.52E-02 | 2.19E-07 | 6.03E-04 | **9.21E-03** | 1.23E-01 | 6.61E-08 | 3.40E-04 |
| ***CDK5RAP2*** | CDK5 Regulatory Subunit Associated Protein 2 | -1.11E-01 | 4.33E-02 | 7.30E-07 | 1.91E-03 | **4.02E-02** | 7.51E-01 | 3.83E-06 | 7.90E-03 |
| ***SC4MOL*** | methylsterol monooxygenase 1 | 1.90E-01 | 7.52E-02 | 1.08E-06 | 2.70E-03 | **2.26E-02** | 2.60E-01 | 1.26E-06 | 3.22E-03 |
| ***HMGCR*** | 3-hydroxy-3-methylglutaryl-coenzyme A reductase | 1.07E-01 | 4.33E-02 | 1.85E-06 | 4.41E-03 | **2.51E-03** | 9.49E-01 | 9.06E-07 | 2.46E-03 |
| *C14orf1* | Chromosome 14 Open Reading Frame 1 | 6.53E-02 | 2.70E-02 | 2.94E-06 | 6.70E-03 | 1.28E-01 | 3.86E-01 | 2.05E-05 | 3.78E-02 |
| *AACS* | acetoacetyl-CoA synthetase | 7.32E-02 | 3.08E-02 | 4.15E-06 | 8.54E-03 | 4.02E-01 | 1.60E-01 | 3.50E-05 | 5.81E-02 |
| ***HMGCR*** | 3-hydroxy-3-methylglutaryl-coenzyme A reductase | 1.02E-01 | 4.29E-02 | 4.23E-06 | 8.54E-03 | **6.97E-03** | 9.61E-01 | 4.80E-06 | 9.51E-03 |
| *FGFBP1* | Fibroblast Growth Factor Binding Protein 1 | 3.33E-01 | 1.40E-01 | 4.24E-06 | 8.54E-03 | 5.15E-02 | 2.95E-01 | 9.95E-06 | 1.90E-02 |
| *DHCR7* | 7-dehydrocholesterol reductase | 1.33E-01 | 5.63E-02 | 4.56E-06 | 8.85E-03 | 1.58E-01 | 6.03E-01 | 5.34E-05 | 7.86E-02 |
| *GINS3* | GINS Complex Subunit 3 (Psf3 homolog) | -9.48E-02 | 4.03E-02 | 5.30E-06 | 9.92E-03 | 5.31E-01 | 8.93E-01 | 2.44E-04 | 2.12E-01 |
| *ELOVL6* | ELOVL Fatty Acid Elongase 6 | 1.84E-01 | 8.09E-02 | 1.08E-05 | 1.88E-02 | 8.42E-01 | 2.40E-01 | 2.16E-04 | 1.98E-01 |
| ***INSIG1*** | insulin induced gene 1 | 1.27E-01 | 5.73E-02 | 1.60E-05 | 2.70E-02 | **8.24E-04** | 6.27E-02 | 1.99E-07 | 7.31E-04 |
| *DHCR7* | 7-dehydrocholesterol reductase | 1.23E-01 | 5.55E-02 | 1.77E-05 | 2.89E-02 | 2.52E-01 | 4.79E-01 | 2.12E-04 | 1.98E-01 |
| *EML1* | Echinoderm Microtubule Associated Protein Like 1 | -1.12E-01 | 5.08E-02 | 2.02E-05 | 3.20E-02 | 5.16E-02 | 5.99E-01 | 7.32E-05 | 1.02E-01 |
| ***INSIG1*** | insulin induced gene 1 | 1.23E-01 | 5.64E-02 | 2.30E-05 | 3.55E-02 | **8.52E-04** | 8.66E-02 | 3.82E-07 | 1.31E-03 |

CI is the confidence interval: the value to add and subtract to the log 2 fold change (Log2FC). BH is the Benjamini-Hochberg adjusted p-values. Genes in bold are replicated in at least one cohort (Groningen). Some genes are represented by more than one transcript.
